# Substance P Alleviates Retinal Pigment Epithelium Dysfunction Caused by High Glucose-Induced Stress

**DOI:** 10.3390/life13051070

**Published:** 2023-04-23

**Authors:** Dahyeon Lee, Hyun Sook Hong

**Affiliations:** 1 Department of Biomedical Science and Technology, Graduate School, Kyung Hee University, Seoul 02447, Republic of Korea; 2 East-West Medical Research Institute, Kyung Hee University, Seoul 02447, Republic of Korea; 3 Kyung Hee Institute of Regenerative Medicine (KIRM), Medical Science Research Institute, Kyung Hee University Medical Center, Seoul 02447, Republic of Korea

**Keywords:** diabetes, oxidative stress, RPE, substance P, inflammation

## Abstract

When the retina is constantly affected by high glucose (HG) due to diabetes, the barrier function of the retinal pigment epithelium (RPE) is impaired, accompanied by unnecessary vascularization. This eventually leads to the development of diabetic retinopathy (DR). This study investigated the recovery effect of substance P (SP) on RPE injured by HG. RPE was treated with HG for 24 h, and HG-induced cellular injuries were confirmed. SP was added to the dysfunctional RPE. Compared to RPE in low glucose (LG) conditions, HG-damaged RPE had large, fibrotic cell shapes, and its cellular viability decreased. HG treatment reduced tight junction protein expression levels and caused oxidative stress by interrupting the antioxidant system; this was followed by inflammatory factor intracellular adhesion molecule-1 (ICAM-1), Monocyte chemotactic protein-1 (MCP-1), and angiogenesis factor vascular endothelial growth factor (VEGF) expression. SP treatment contributed to RPE recovery by enhancing cell viability, tight junction protein expression, and RPE function under HG conditions, possibly by activating the Akt signaling pathway. Importantly, SP treatment reduced ICAM-1, MCP-1, and VEGF expression. Collectively, SP activated survival signals to suppress oxidative stress and improve retinal barrier function in RPE, accompanied by immune suppression. This suggests the possible application of SP to diabetic retinal injuries.

## 1. Introduction

Diabetic retinopathy (DR) is a microvascular disorder and a common complication of diabetes. DR is characterized by edema and neovascularization in the retinal layer, leading to disastrous consequences of visual impairment [1]. DR is classified into two types, non-proliferative diabetic retinopathy (NPDR) and proliferative diabetic retinopathy (PDR). NPDR alters the blood vessels responsible for nutrient supply to the retina, which interrupts the physiological function of the blood vessels and induces exudate or bleeding. If vascular dysfunction is not treated early, it can develop into PDR, and new blood vessels are aberrantly formed [2]. This increases retinal degeneration, leading to vision loss.

The outer pigmented layer of the retina is the retinal pigment epithelium (RPE). The RPE is a polarized monolayer that separates the inner layer of photoreceptors from the choroid and plays an essential role in the structural and functional homeostasis of the retina [3,4]. Thus, RPE acts as an external blood–retinal barrier (BRB) to protect and maintain photoreceptors [5,6,7,8,9]. RPE is actively involved in the uptake of a range of nutrients, including glucose, retinol, fatty acids, and ascorbic acid, from the blood and regulates the transport of these nutrients to the photoreceptors [10,11,12]. RPE cells are also involved in defensive immune mechanisms of the macula, phagocytosis of photoreceptor outer segments, maintenance of ocular angiogenic balance, and preservation of their phototransduction [10,12,13,14]. Considering the critical role of RPE, failure of RPE function is strongly associated with major ocular clinical alterations, including retinal degeneration and irreversible vision loss [15].

The maintenance of high blood glucose due to diabetes can break the cellular junction, the main component of the BRB, and increase RPE permeability [16,17]. Moreover, high glucose levels cause GLUT1 down-regulation in the RPE as well as reduced antioxidant levels, potentially leading to retinal tissue damage [3]. Under these circumstances, RPE generates inflammatory factors, such as interleukin (IL)-6, IL-8, IL-1β, monocyte chemoattractant protein (MCP-1), and vascular endothelial growth factor (VEGF) [18,19]. Upregulation of these paracrine factors induces permanent retinal damage by inducing RPE apoptosis, excessive inflammation, and retinal neovascularization [20]. If this condition is sustained without treatment, DR eventually develops.

Various clinical treatments have been developed to relieve the symptoms of DR. First, many therapeutics target the survival or function of photoreceptors [6]. Selective inactivation of the insulin receptor and pyridoxamine, which are involved in vitamin B production, can inhibit photoreceptor loss [21,22]. Fibroblast growth factor 21 can protect photoreceptor function [23], and pituitary adenylate cyclase-activating polypeptide can ameliorate photoreceptor degeneration [24]. As a treatment to protect the RPE barrier, anti-VEGF is widely used to prevent neovascularization with high efficacy [7,25]. Laser photocoagulation and vitrectomy are also used to delay vision impairment [26]. In some diseases, such as age-related macular degeneration (AMD) and DR, RPE transplantation has been explored [27,28]. Recently, targeting specific signaling factors in DR using miRNA or stem cell transplantation for RPE regeneration has also been attempted [29,30]. However, the application of these therapies is limited because of their weak efficacy and high manufacturing costs. Moreover, most treatments can slow the progression of diabetic retinopathy but cannot recover the RPE.

Substance P (SP; RPKPQQFFGLM) is an 11 amino acid endogenous neuropeptide that preferentially binds to the neurokinin 1 receptor (NK-1R), and it is expressed on various cell types including neuronal cells, immune cells, endothelial cells, and adult stem cells. SP is known to be involved in vasodilation and cell proliferation, migration, and survival. Additionally, the systemic application of SP was found to stimulate mobilization of bone marrow mesenchymal stem cells to the circulation by repopulating mesenchymal stem cell within bone marrow environment [31]. SP is able to promote the transition of M1 monocyte to M2 type under the acute spinal cord injury, leading to suppression of inflammatory response and the accelerated repair [32]. SP was also reported to suppress inflammation-induced endothelial injury by elevating level of the eNOS/NO [33]. Previous studies have confirmed that SP can ameliorate cell injury in a variety of cells by restoring cellular structure/function under pathological condition [34,35,36,37,38]. SP treatment prevents fibrotic changes in the RPE by regulating tumor necrosis factor-α (TNF-α), attributable to inflammatory conditions in BRB [38]. Additionally, SP modulates Akt/GSK-3β signaling to protect RPE against stress from hydrogen peroxide [37]. Given the role of SP studied so far, SP was hypothesized to block high glucose-induced damage in the RPE, possibly by inhibiting cellular damage, inflammation, and oxidative stress.

The goal of this study was to investigate whether SP protects the RPE against a high glucose-induced pathological environment. To mimic the diabetic environment in vitro, RPE cells were exposed to high glucose levels using ARPE-19 and the human retinal pigmented epithelial cell line, and cellular alterations were analyzed in terms of cell viability and loss of structural/functional molecules over time. After the confirmation of RPE impairment due to high glucose levels, the RPE was treated with SP. The restorative effect of SP was determined by analyzing cell viability, proliferation rate, tight junction expression, and secretion of inflammatory/angiogenic factors in ARPE-19 cells in the presence of high glucose.

## 2. Materials and Methods

### 2.1. Materials

ARPE-19 cells were purchased from ATCC (Manassas, VA, USA). Dulbecco’s modified Eagle’s medium (DMEM) and fetal bovine serum (FBS) were obtained from Gibco (Grand Island, NY, USA). Phosphate-buffered saline, penicillin/streptomycin, and trypsin-ethylenediaminetetraacetic acid were purchased from Welgene (Daegu, Republic of Korea). SP and phenylmethylsulfonyl fluoride (PMSF) were obtained from Sigma–Aldrich (St. Louis, MO, USA). Anti-GAPDH, anti-ICAM-1, and anti-RPE65 antibodies (Abcam, Cambridge, MA, USA) were also used. Anti ZO-1, anti E-cadherin (24E10), anti-Akt, and anti-phospho-Akt (ser473) antibodies and cell lysis buffer were purchased from Cell Signaling Technology (Danvers, MA, USA).

### 2.2. Cell Culture

ARPE-19 cells were grown in 1 g/L glucose DMEM containing 10% FBS and 100 U/mL penicillin/streptomycin at 37 °C with 5% CO_2_. The culture medium was changed every alternate day. Images were obtained using a microscope (Nikon Eclipse; Tokyo, Japan). When cell confluency was almost 80%, the culture medium was replaced with fresh medium containing 1 g/L (low glucose, LG) or 4.5 g/L (high glucose, HG) glucose. THP-1 (ATCC) was cultured in RPMI 1640 medium 10% FBS and 1% penicillin/streptomycin.

### 2.3. SP Administration

SP powder was solubilized in saline. SP treatment was carried out 24 h post-high glucose treatment at a final dose of 100 nM by adding to the conditioned medium. Saline was used as a control.

### 2.4. MTT Assay

To determine cell viability, the MTT (3-(4,5-Dimethylthiazol-2-yl)-2,5-Diphenyltetrazolium Bromide) solution was added to each well. After incubation at 37 °C for 3 h, the supernatant was removed, and formazan crystals were dissolved in Dimethyl sulfoxide. Absorbance was measured at 570 nm using a microplate reader (Molecular Devices, San Jose, CA, USA). The cellular activity under each experimental condition is expressed as a percentage relative to the activity of the LG group.

### 2.5. Western Blotting

ARPE-19 cell lysates were obtained using a lysis buffer/2 mM phenylmethylsulfonyl fluoride solution (PMSF). The protein concentration was determined using a BCA Protein assay kit (Thermo Fisher Scientific, Rockford, IL, USA). Lysates were denatured, and sodium dodecyl sulfate polyacrylamide gel electrophoresis was carried out. The proteins were transferred to nitrocellulose membranes then the membrane was blocked with 5% skim milk and incubated with the primary antibodies. After washing with tris-buffered solution containing 0.1% Tween 20, the membrane was exposed to horseradish peroxidase-conjugated secondary antibodies at room temperature. Membranes were developed using WESTAR ETA C ULTRA 2.0, (Cyanagen, Bologna, Italy) and visualized using Vilber imager chemiluminescence (Vilber, Collégien, France). Expression levels were relatively quantified using ImageJ software.

### 2.6. Glutathione Peroxidase (GPx) Activity Assay

ARPE-19 cells were lysed using a lysis buffer/2 mM PMSF solution. After determining the protein concentration, GPx activity was assessed, according to the manufacturer’s protocol (GPx Activity Assay Kit; Abcam, Cambridge, MA, USA).

### 2.7. Enzyme-Linked Immunosorbent Assay (ELISA)

Conditioned media for ELISA were collected by centrifugation at 1500 rpm for 5 min at 4 °C. Human MCP-1 and VEGF concentration was measured, according to the manufacturer’s instructions (VEGF, MCP-1 ELISA kit; R&D Systems, Minneapolis, MN, USA). In brief, all reagents, standard dilutions, and samples were prepared and added to primary antibody-coated well as directed for 2 h at RT, followed by washing four times. Next, the horseradish peroixdase-conjuated secondary antibody solution was treated to each well, and each well was repeatedly washed. Two h later, substrate solution was added. Once the color of the solution changed to blue, the reaction was stopped, and the optical density was measured with the wavelength correction set to 450 nm using an Emax Endpoint ELISA Microplate Reader (Molecular Devices, Sunnyvale, CA, USA). The quantification was completed based on the standard.

### 2.8. Transwell Migration Assay

Cell culture insert (5 um pore size) was put in 24 well tissue culture plate (Corning Co., Corning, NY, USA). THP-1 suspension in culture media was seeded in the insert (200 μL), and a mixture of RPMI 1640 and RPE conditioned medium was added to the bottom of 24 well plate. Five h later, the insert was removed, and migrated cells were counted with trypan blue staining.

### 2.9. Statistical Analysis

All data are presented as mean ± standard deviation. Statistical analyses were performed using an unpaired two-tailed Student’s *t*-test in GraphPad Prism (GraphPad Software, San Diego, CA, USA). *p* < 0.05 was set as the boundary of statistical significance (* *p* < 0.05, ** *p* < 0.01, *** *p* < 0.001).

## 3. Results

### 3.1. High Glucose Reduces Cellular Activity and Proliferation Rate of RPE

**Figure 1:**
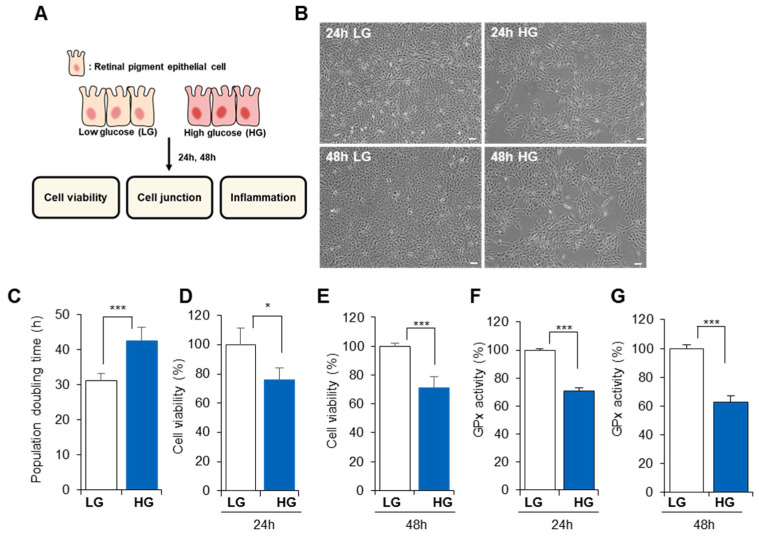
High glucose levels decrease RPE viability and proliferation rate. (**A**) The effect of high glucose levels on ARPE-19 was analyzed over time. (**B**) The cellular shape of RPE was monitored for 48 h after low glucose (LG) or high glucose (HG) treatment. Scale bar: 100 μm (**C**) Proliferation rate was assessed by calculating the mean population doubling time over 24 and 48 h. Cellular viability was determined by MTT assay at 24 (**D**) and 48 h (**E**). GPx activity was determined at 24 (**F**) and 48 h (**G**). Values represent the mean ± standard deviation of three independent experiments. * *p* < 0.05, *** *p* < 0.001.

To create diabetes-induced cellular stress, RPE cells were exposed to HG in vitro, and cellular alterations were observed for 48 h (Figure 1A). LG-exposed RPE maintained a small, round cell shape with a compact cellular junction, whereas HG-exposed RPE showed a reduced proliferation rate and large, fibrotic cell shape (Figure 1B). The detrimental effect of HG on cellular shape was observed at 24 h post-treatment and was sustained for 48 h without cellular restoration.

Compared to LG-exposed RPE, HG treatment for 48 h induced a decreased RPE proliferation rate (Figure 1C, population doubling time (PDT) in LG: 31.2 ± 2.1 h; PDT in HG: 42.5 ± 3.9 h, *p* < 0.001). Cell viability analysis revealed that HG treatment clearly reduced RPE viability within 24 h; this phenomenon was maintained until 48 h post-HG exposure (Figure 1D, cell viability in HG for 24 h: 76.18 ± 7.84%; Figure 1E, cell viability in HG for 48 h: 71.1 ± 7.5%). HG treatment destroys the ROS-regulating system and induces oxidative stress in cells [39]. GPx protects organisms from oxidative damage, and its loss is closely related to the development of oxidative stress. In this experiment, GPx activity was clearly reduced by HG treatment within 24 h and sustained for 48 h (Figure 1F,G; GPx in HG for 24 h: 70.73 ± 2.43%; Figure 1E, GPx in HG for 48 h: 62.55 ± 4.51%).

These data suggest that HG conditions cause cellular injury in the RPE by disturbing cell viability, antioxidant activity, and repopulation potential. This implies a deficiency in the restorative capacity of the RPE under diabetic conditions.

### 3.2. High Glucose Levels Alter RPE Cellular Characteristics

**Figure 2:**
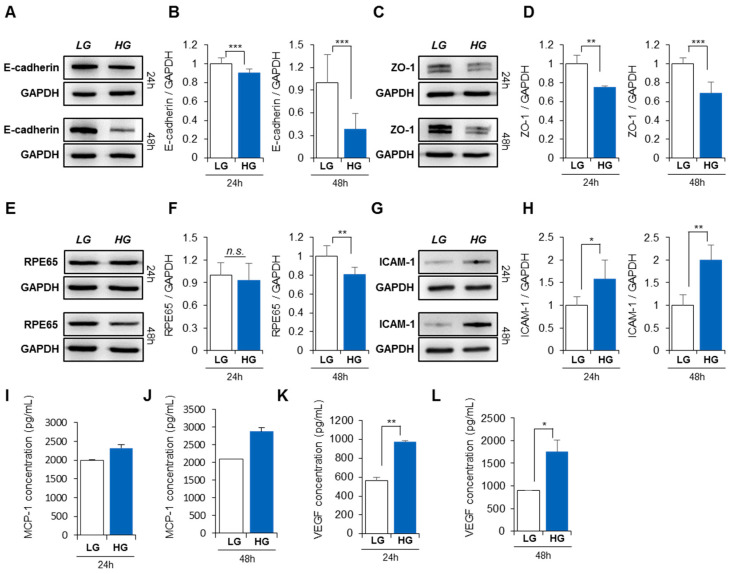
RPE cellular characteristics are altered by high glucose levels. RPE cells were lysed for western blotting, and E-cadherin and ZO-1 expression levels were examined at 24 (**A**,**C**) and 48 h (**B**,**D**). Protein levels of RPE65 were evaluated at 24 (**E**) and 48 h (**F**) after LG or HG treatment. ICAM-1 expression was assessed at 24 (**G**) and 48 h (**H**). MCP-1 and VEGF production were analyzed by ELISA at 24 (**I**,**K**) and 48 h (**J**,**L**). Values represent mean ± standard deviation of three independent experiments. * *p* < 0.05, ** *p* < 0.01, *** *p* < 0.001.

RPE is a type of epithelial cell with tight junctions, and its main role is to act as a barrier to distinguish photoreceptors from the choroid and inhibit choroidal neovascularization. To maintain a compact layer of the RPE, its junction structure should be tightly preserved. As shown in Figure 1B, HG treatment induced morphological changes in the RPE to a fibroblastic shape. The transition of epithelial cells to a mesenchymal/fibrotic shape may include the loss of epithelial-specific junction molecules, which are essential for a compact layer. To examine the effect of HG on cellular junctions of RPE, epithelial cadherin (E-cadherin) and zonula occludens-1 (ZO-1) expression levels were analyzed by western blot. HG treatment for 24 h firmly reduced E-cadherin levels (Figure 2A) and ZO-1 expression (Figure 2C) in RPE, and this effect was more evident 48 h post-HG (Figure 2B,D). This indicates that HG conditions can induce a lack of barrier function in the RPE and destroy the retinal layer.

RPE65 is an enzyme that regulates the retina in the visual cycle and represents RPE function. There was little change in RPE65 expression after 24 h of exposure to HG (Figure 2E), but reduced expression was observed at 48 h (Figure 2F), implying structural alteration of the RPE, followed by functional loss in the RPE under HG conditions.

Retinal inflammation is a characteristic of DR. Many reports have shown that diabetes enhances inflammatory factor expression in the RPE [40,41,42], and this environment is related to the development of retinal degeneration. Of the inflammation-related factors, ICAM-1, which enhances leukocyte recruitment, is markedly elevated under diabetic conditions [42]. In this study, ICAM-1 expression was determined under HG conditions. As predicted, HG exposure increased ICAM-1 expression within 24 h, which was exacerbated 48 h after HG treatment (Figure 2G,H). As the main chemoattractant, MCP-1 is clearly elevated by HG. MCP-1 quantification revealed that HG treatment promoted MCP-1 secretion at 24 h, and its impact was intensified at 48 h (Figure 2I,J).

HG-induced breakdown of the RPE layer and inflammatory conditions permit neovascularization of the retinal layer. The breakdown of tight junctions in the RPE was found to promote vascular leakage, as observed in diabetic and ischemic rodents [43]. Thus, clinical treatment aims to block vessel formation or relieve inflammation in patients with DR. VEGF plays a significant role in neovascularization and can be a target to inhibit retinal vascularization in patients with DR. To check if HG conditions affect VEGF secretion in the RPE, VEGF concentration in the RPE was quantified by ELISA. RPE treated with HG for 24 h produced more VEGF than LG-treated RPE, and VEGF production was elevated after exposure to HG for 48 h (Figure 2K,L).

These results confirm that HG conditions can impair cellular structure and function and create an inflammation-favorable environment in the retina.

### 3.3. SP Prevents Reduction of RPE Activity Due to High Glucose Levels

**Figure 3:**
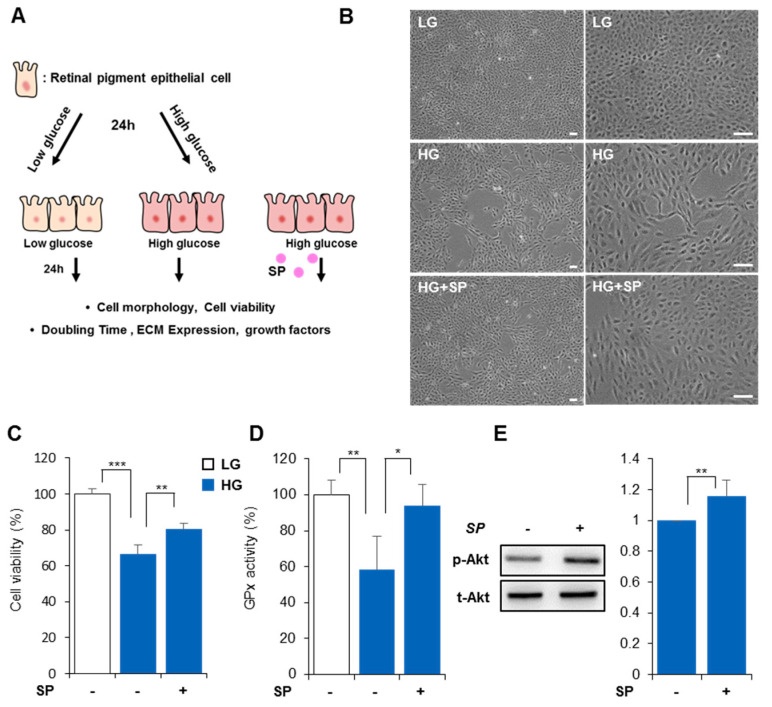
Substance-p protects RPE cellular activity from high glucose levels. (**A**) SP was added to RPE exposed to HG for 24 h, and cellular activity was analyzed 48 h post-HG treatment. (**B**) At 48 h after HG exposure, the cellular shape of RPE was observed. Scale bar: 100 μm. (**C**) Cell viability was evaluated at 48 h by MTT assay. (**D**) Oxidative stress was assessed by measuring GPx activity. (**E**) Relative p-AKT expression was evaluated by western blotting. Values represent mean ± standard deviation of three independent experiments. * *p* < 0.05, ** *p* < 0.01, *** *p* < 0.001.

HG conditions mostly caused cellular impairment within 24 h, which was maintained for 48 h. To investigate the effect of SP on impaired RPE, SP was administered to RPE pretreated with HG for 24 h. The effects were assessed 24 h after SP treatment (Figure 3A). Compared to LG-treated RPE, the HG-treated RPE had a low repopulation rate. However, SP treatment ameliorated the RPE cell proliferation rate. Regarding cellular morphology, the LG-treated RPE had a normal appearance with small, round shapes. However, HG-treated RPE showed fibrous morphology, which was clearly alleviated by SP treatment (Figure 3B). HG treatment reduced RPE cell viability, but SP treatment improved cell viability in HG within 24 h (Figure 3C, cell viability in HG: 66.2 ± 5.4%; cell viability in HG + SP: 80.48 ± 3.01%, *p* < 0.05).

Diabetic retinopathy is accompanied by oxidative stress and inflammation [44]. When RPE was exposed to HG for 48 h, GPx activity declined (Figure 3D), but SP treatment inhibited this reduction (GPx activity in HG-treated RPE: 58.3 ± 18.9%; HG+SP-treated RPE: 93.9 ± 11.7%). This might be related to the SP-mediated increase in cell viability because excessive ROS accumulation due to a lack of an antioxidant system can affect cell viability.

Cell survival-related signaling [45], such as Akt signaling, should be activated to enhance cell viability. Pathological stress, including oxidative stress, is ameliorated by Akt signaling pathway activation [45,46]. A previous study found that SP can enhance cell viability through the Akt signaling pathway under pathological conditions [36]. To examine whether SP-enhanced cellular activity and antioxidant activity are accompanied by Akt signaling or not, phospho-Akt and total Akt expression were analyzed 10 min after SP treatment under HG conditions. As shown in Figure 3E, SP induced Akt phosphorylation, which is anticipated to contribute to the restoration of cell viability in RPE under HG conditions (Figure 3E).

These data suggest that SP can prevent impairment of the RPE due to HG by modulating oxidative stress and cellular viability.

### 3.4. SP Mitigates Functional/Structural Deficiency of RPE from High Glucose-Induced Oxidative Stress

**Figure 4:**
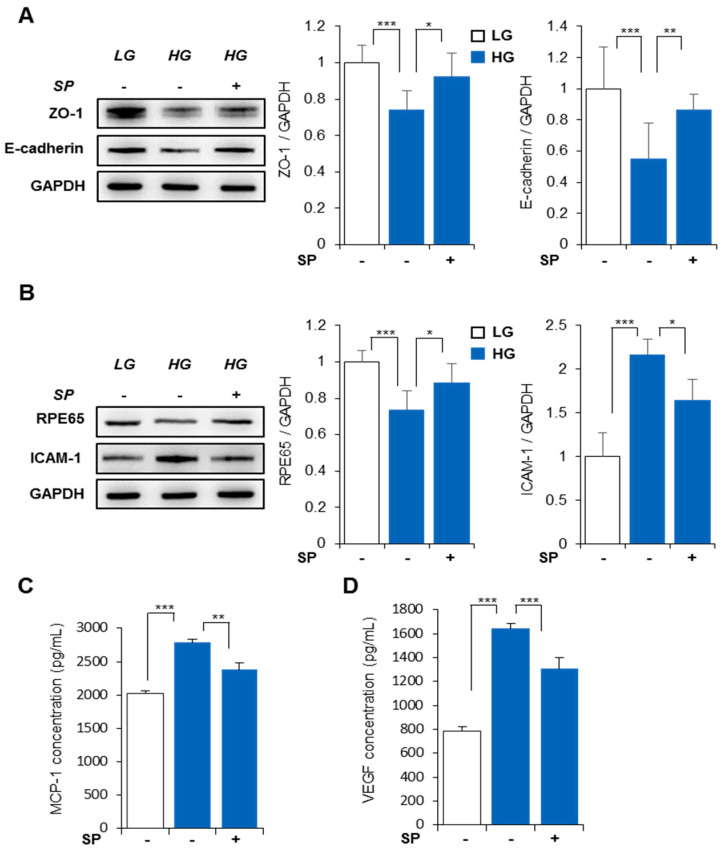
SP ameliorates alteration of high glucose-damaged RPE. SP was added to RPE cells pre-treated with HG for 24 h. Cell lysates were prepared 24 h later for analysis. (**A**) ZO-1 and E-cadherin expression levels were analyzed by western blotting. (**B**) RPE65 and ICAM-1 expression were assessed by western blotting. (**C**,**D**) MCP-1 and VEGF secretion were evaluated in conditioned media by ELISA. Values represent mean ± standard deviation of three independent experiments. * *p* < 0.05, ** *p* < 0.01, *** *p* < 0.001.

As shown in Figure 2, HG treatment induced alteration of RPE by decreasing ZO-1/E-cadherin expression and causing reduction of RPE65 expression. Next, to determine the effect of SP on tight junction proteins in RPE under HG conditions, ZO-1 and E-cadherin expression was examined 24 h post-SP treatment. SP treatment distinctly inhibited the HG-mediated loss of ZO-1 and E-cadherin (Figure 4A). Moreover, SP inhibited RPE65 loss under HG stress (Figure 4B); i.e., treatment is anticipated to be able to contribute to BRB maintenance by preserving the RPE layer in diabetic conditions.

Oxidative stress provokes inflammatory responses [35,37,38]. As shown in Figure 2B, ICAM-1 expression clearly increased 48 h after HG treatment, consistent with the previous reports. However, ICAM-1 expression clearly decreased with the addition of SP. Moreover, SP treatment decreased MCP-1 production in RPE cells under HG conditions (Figure 4C). This indicates that SP is able to ameliorate HG-induced immune cell recruitment in the RPE. Moreover, new VEGF-induced blood vessels disturb normal circulation by inducing bleeding in the body in diabetes. As shown in Figure 4D, HG markedly increased VEGF production compared to LG, indicating undesired/excessive vessel formation. However, SP treatment ameliorated VEGF secretion under HG conditions (Figure 4D).

Collectively, these results confirm that SP prevents RPE deterioration by preserving RPE structure and function and also prevents an inflammation and aberrant vasculature-enriched environment forming by modulating paracrine factors from RPE in diabetes.

### 3.5. SP Treatment Modulates Paracrine Action of RPE under HG Condition

**Figure 5:**
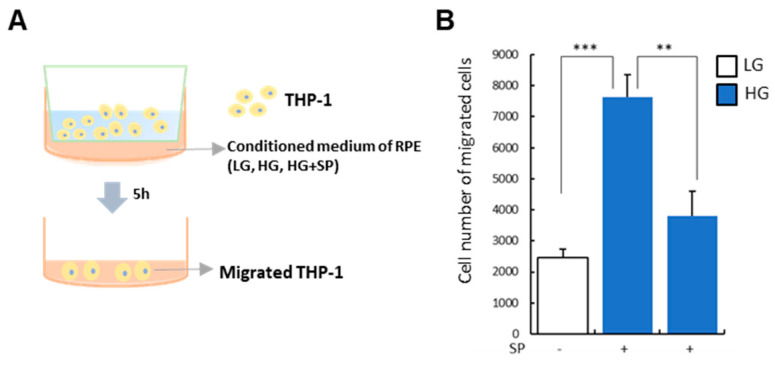
SP treatment suppresses RPE-mediated immune cell migration. RPE was cultured in each condition and its conditioned medium (CM) was added to THP-1 in transwell. 5 h later, migrated cell into the bottom was compared. (**A**) Diagram of CM treatment (**B**) Migrated cell from upper to the bottom was counted. Values represent mean ± standard deviation of three independent experiments. ** *p* < 0.01, *** *p* < 0.001.

HG condition destroys RPE structure by disturbing cellular junction. Moreover, maintenance of HG could make cellular state of RPE favorable for inflammatory cells migration and attachment by elevating the generation of ECM and chemokines. HG treatment elevated MCP-1 for recruitment and ICAM-1 for attachment in RPE, which was suppressed by SP treatment. Next, in order to check the paracrine action of RPE for inflammatory cells under HG, the conditioned medium (CM) of RPE was treated with THP-1 and the human monocyte cell line, and its migratory capacity was evaluated in vitro (Figure 5A). Compared to CM of LG-treated RPE, CM of HG-treated RPE rarely activated THP-1 but obviously facilitated its migration. However, its migratory effect was relieved by CM from SP-treated RPE under the HG condition (Figure 5B). Although SP treatment did not reverse the state of RPE into the normal (LG-treated), the presence of SP certainly blocked elevation of MCP-1 (Figure 4) to maintain low level of MCP-1. Thus, the reduction of MCP-1 by SP is expected to contribute to the decline of the migration of THP-1.

If inflammatory factors are reduced in the retina, immune cell infiltration into tissue and local tissue damage would decline. Thus, these data revealed that SP treatment can alleviate inflammatory stress attributable to soluble factors from RPE under the diabetes, and furthermore, SP treatment might inhibit development of DR or slow down the progression of retina disease.

## 4. Discussion

RPE acts as a significant barrier that protects the photoreceptor by controlling the exchange of materials between the outer retina and choriocapillaris. As the RPE exists between the photoreceptor and the choroid as a monolayer, it may encounter a highly oxidative microenvironment under pathological conditions. If the RPE is damaged and destroyed in aspects of the structure and function, homeostasis of the retina is entirely disrupted [44], leading to serious retina disease with vision loss. Thus, the compact layer of the RPE should be protected to prevent the development of serious retinal disease including DR, proliferative vitreoretinopathy, or AMD [47].

Maintenance of excessive glucose causes several dysmetabolic processes, leading to oxidative stress [48]. Previous studies have shown that ROS generation leads to RPE apoptosis, epithelial-mesenchymal transition, DNA damage, decreased proliferation rate, choroidal neovascularization, and inflammation [37,48,49,50,51,52,53,54]. Therefore, numerous antioxidants have been suggested to preserve the RPE layer [55]. This is consistent with the increased oxidative stress observed under high glucose conditions [12]. Phagocytosis is a major function of RPE cells that removes excess photoreceptor outer segments. High-glucose conditions result in decreased phagocytic activity of RPE cells, which may make an inflammatory environment in retina because of the lack of the clearance. As is well known, microvascular modification is a critical problem associated with diabetic complications. Alterations in the blood vessels are inevitably fatal for photoreceptors in the retina [56]. RPE, which is therefore deficient in nutrients and oxygen due to vascular obstruction, requires the formation of new blood vessels for retina homeostasis. These new microvessels have aberrant structures, unlike normal, and rapidly induce deformation of the retina including retina folding and detachment, which might cause diabetes-mediated retinal vascular dysfunction. This can be the direct cause of vision loss and intensifies retinal inflammation. Thus, attempts to block vascularization and inflammation progression have been constantly attempted in patients with DR. VEGF is considered a representative factor that initially creates new blood vessels. Thus, in clinic, steroids to suppress inflammation and anti-VEGF antibodies to inhibit vessel formation are constantly applied, but their use is rigorously controlled because of the side effects and high cost. Long-term treatment with steroids evokes elevated intraocular pressure and cataracts. The injection method can cause complications including endophthalmitis, vitreous hemorrhage, and retinal detachment. The necessity for new treatment is obvious, but therapeutic strategy should be discovered.

This study has explored cellular change by HG in vitro. It reveals that the HG condition reduced the expression of the junction proteins ZO-1 and E-cadherin, resulting in a structural lack of RPE. Simultaneously, HG conditions reduced cell viability, repopulation capacity, and RPE65 levels to interrupt the regenerative potential of RPE. HG treatment decreased GPx activity and upregulated ICAM/MCP-1 expression, accompanied by VEGF-enriched conditions, as previously demonstrated. This implies that constant stress from HG impairs both the structure and function of the RPE with inflammation/vascularization, resulting in retinal detachment and degeneration.

SP was reported to inhibit ROS-induced cell death and inflammation and restores cellular activity [35,37,57]. However, the study for the effect of SP on RPE cells under the diabetes was deficient. This study aimed to determine whether SP can affect dysfunctional RPE due to HG-induced oxidative stress in cellular/molecular level. The expression of NK-1R on RPE was previously confirmed. SP treatment was performed 24 h after exposure to HG. SP treatment blocked the reduction in RPE viability and proliferation caused by HG stress. SP-treated RPE sustained higher expression levels of junction molecules and higher antioxidant activity than non-treated RPE. Analysis of early survival-related signaling showed that SP activated Akt signaling in impaired RPE. Importantly, SP treatment blocked HG-mediated ICAM-1 expression and MCP-1 production. This effect was confirmed by examining the paracrine effect of SP-treated RPE on monocyte migration capacity. That is, SP treatment can reduce retinal inflammation by blocking acquirement of inflammatory features in RPE under diabetic conditions. Moreover, SP relieved the HG-induced increase in VEGF production due to HG, suggesting that SP can reduce unnecessary vessel formation in the retina. Control of inflammation and vascularization by SP is expected to be able to be a potential new solution to prevent DR progression.

## 5. Conclusions

The exposure of RPE cells to HG resulted in a significant increase in oxidative stress, inflammation, and cellular impairment. However, SP was able to rejuvenate HG-damaged RPE cells by modulating oxidative stress, inflammatory factors, angiogenic factors, and survival signaling pathways. This suggests that SP has the potential to treat DR by targeting the RPE. Testing of the safety of systemically applied SP has been completed in non-clinical studies [58,59], and this can increase the possibility to develop SP as a medical drug. As further study, the efficacy of SP should be explored in a non-clinical animal model.

## Data Availability

The datasets used and/or analyzed during the present study are available from the corresponding author upon reasonable request.
